# Long-term genetic monitoring of a riverine dragonfly, *Orthetrum coerulescens* (Odonata: Libellulidae]: Direct anthropogenic impact versus climate change effects

**DOI:** 10.1371/journal.pone.0178014

**Published:** 2017-05-26

**Authors:** Rebecca Herzog, Heike Hadrys

**Affiliations:** 1 ITZ, Ecology & Evolution, University of Veterinary Medicine Hannover, Hannover, Germany; 2 American Museum of Natural History, The Sackler Institute for Comparative Genomics, New York, New York, United States of America; 3 Department of Ecology and Evolutionary Biology, Yale University, New Haven, Connecticut, United States of America; National Cheng Kung University, TAIWAN

## Abstract

Modern conservationists call for long term genetic monitoring datasets to evaluate and understand the impact of human activities on natural ecosystems and species on a global but also local scale. However, long-term monitoring datasets are still rare but in high demand to correctly identify, evaluate and respond to environmental changes. In the presented study, a population of the riverine dragonfly, *Orthetrum coerulescens* (Odonata: Libellulidae), was monitored over a time period from 1989 to 2013. Study site was an artificial irrigation ditch in one of the last European stone steppes and “nature heritage”, the Crau in Southern France. This artificial riverine habitat has an unusual high diversity of odonate species, prominent indicators for evaluating freshwater habitats. A clearing of the canal and destruction of the bank vegetation in 1996 was assumed to have great negative impact on the odonate larval and adult populations. Two mitochondrial markers (CO1 & ND1) and a panel of nuclear microsatellite loci were used to assess the genetic diversity. Over time they revealed a dramatic decline in diversity parameters between the years 2004 and 2007, however not between 1996 and 1997. From 2007 onwards the population shows a stabilizing trend but has not reached the amount of genetic variation found at the beginning of this survey. This decline cannot be referred to the clearing of the canal or any other direct anthropogenic impact. Instead, it is most likely that the populations’ decay was due to by extreme weather conditions during the specific years. A severe drought was recorded for the summer months of these years, leading to reduced water levels in the canal causing also other water parameters to change, and therefore impacting temperature sensitive riverine habitat specialists like the *O*. *coerulescens* in a significant way. The data provide important insights into population genetic dynamics and metrics not always congruent with traditional monitoring data (e.g. abundance); a fact that should be regarded with caution when management plans for developed landscapes are designed.

## Introduction

Due to increasing anthropogenic impact on natural ecosystems the number of species that call for conservation management grows steadily [1]. Long-term genetic monitoring data could provide a better understanding of human impact on species and their habitats. Nevertheless time-series studies especially on local scales are still rare. Although the term ‘genetic monitoring’ has been established in all areas of research concerning evolution, ecology and conservation, it is still not commonly defined regarding the temporal dimension [2]. In this study we use it for the observation of population genetic parameters over time, rather than an assessment providing a snapshot of certain features for a given point in time.

Genetic monitoring provides important insights into population genetic metrics and offers an over-time evaluation [2]. Only long-term data sets can reliably distinguish between sampling error and reasonable changes in abundance, population genetic metrics or influence of environmental variability [3, 4]. Even more important, changes in genetic variation—the raw material of evolution—affect a population’s viability over time [2]. Consequently monitoring genetic diversity over a certain time period offers the chance to foresee trends and detect populations in need of conservation measures [2]. Particularly studies of anthropogenic influence on a regional scale are missing as conservationists call for large scale global assessments.

In this study one of the largest populations of the libellulid dragonfly *Orthetrum coerulescens* in France was genetically monitored since 1989. Odonata (dragonflies & damselflies) with their complex life cycles—aquatic larvae and terrestrial imagos—are important model systems for evaluating freshwater ecosystems worldwide [5, 6]. They show a wide range of habitat specificity from generalists to specialists and are further of increasing conservational interest. The order includes many endangered species, most likely because of their sensitivity to environmental changes [7].

Riverine habitats are especially prone to anthropogenic influences. The main driver of loss of biodiversity in this case is land use, as modelling data show [8]. Dredging, canalization and siltation of streambeds are common threats to species inhabiting running water systems in developed landscapes. The studied species *O*. *coerulescens* has a high specificity to small running waters and fens e.g. [9]. It is red listed in Germany and other Northern European countries but larger populations are still recorded from Southern Europe [10, 11]. One of the largest populations can be found in Southern France, inhabiting a small irrigation ditch in the Crau, a prominent regional diversity hotspot for Odonata.

The Crau is a unique ecosystem due to its extreme ecological conditions. Here, the dry Mediterranean climate (500 - 600mm precipitation per year, 14.5°C annual mean temperature [12]) is combined with exceptional soil characteristics. In 40-60cm depth, a water impermeable conglomerate layer prevents recruitment of ground water [13]. Hence hot and dry, the Crau is representing one of the last xeric steppes in Europe [13]. This grassland provides home for endangered and endemic species [14, 15]. It is, however, highly threatened by land use and intensive agricultural efforts over decades [15, 16]. Numerous artificial irrigation ditches were built to use large parts of this dry area for fruit cultivation and hay production, altering its ecosystem irreversibly [15, 16]. The study object, the Canal de Vergières (CdV) is still in agricultural usage. It is managed by dredging and cutting off the bank vegetation frequently to maintain flow velocity and provide free access to grazing sheeps. This should have a large impact on odonate larval as well as adult populations which are highly specialized and restricted to the characteristics of the sediment, chemistry and bank vegetation of their habitats [17]. Nevertheless as it seems, an unexpected high odonate species richness has been observed here over decades [18], most likely by offering an important freshwater habitat within this arid ecosystem.

This study aims to detect possible genetic consequences that could be referred to direct anthropogenic impact, especially a clearing of the channel in 1996. We monitored the largest CdV dragonfly population, the *Orthetrum coerulescens* population, as an indicator species for this vulnerable freshwater habitat. Hereby the focus is on the influence of the dredging and the population’s potential to recover from its effect. A panel of microsatellite loci developed by Hadrys *et al*.[19], as well as two mitochondrial gene fragments are used in this study: ND1 (NADH Dehydrogenase Subunit 1) and CO1 (Cytochrome C Oxidase Subunit 1) served as approved markers on the population level [20, 21]. With this first long(er) term study of population genetic metrics of a sensitive freshwater insect species it might be possible to distinguish between a populations snapshot and reliable dynamics related to environmental changes and/or anthropogenic impact.

## Material and methods

### Field study and sampling site

Study habitat is the *Canal de Vergières* (CdV) in southern France, located in the “nature heritage”, the Crau (43°36'17.7"N 4°48'34.4"E). Together with the adjacent Camargue, the Crau is one of Europe’s richest odonate biotopes [19]. Decades of non-protection of this region resulted in its extensive use for agriculture. Many irrigation ditches were built cutting through this region. One of them, the CdV, is 13km long and famous for its remarkable odonate species richness. It is the breeding site for at least 32 dragonfly species [22] and has been considered a study site for intensive research on dragonflies and damselflies in the past [20, 23–25]. For the monitoring a 1.5 km long segment in the nature reserve Peau de Meau (belonging to the nature reserve Coussouls de Crau) was chosen. Here, we expected the most stable conditions due to the conservation status of this specific part of the channel. From the beginning of our monitoring in 1989 until 1995 the habitat structure of the CdV remained nearly undisturbed. During the winter months of 1996 the sediment of the ditch was dredged out and all bank vegetation was destroyed in order to maintain flow velocity.

For the genetic monitoring of the *O*. *coerulescens* population tissue samples of 285 adult individuals were collected over a period from 1989 up to 2013 (see Table 1) using the non-invasive sampling method of Fincke & Hadrys [26] where animals were released afterwards. Collection permits were kindly granted from the RNN des Coussouls de Crau. These samples were stored at -20°C until analysis.

**Table 1:** Genetically analyzed tissue samples of *Orthetrum coerulescens* at the CdV in southern France.

Species	Country	Locality	Year	ND1 *N Seq*.	CO1 *NSeq*.	Microsatellites [*N Alleles*]
*O*. *coerulescens*	France	CdV	1989	16	5	49
			1998	24	--	76
			2004	22	4	49
			2007	12	5	12
			2008	17	8	17
			2009	17	10	--
			2010	18	8	24
			2011	21	--	23
			2012	20	17	--
			2013	18	15	--
				∑195	∑ 84	∑250

### Sequence analyses

Genomic DNA was extracted from a single midleg of each individual following a standard phenol-chloroform protocol by Hadrys *et al*. [27]. Two mitochondrial gene fragments were amplified: the ND1 (NADH dehydrogenase subunit 1) fragment, including partial 16S rRNA and tRNALeu, as well as the CO1 (Cytochrom oxidase subunit 1) fragment for which the primer pair OdoCO1 fw 5´>TAC ACG AGC ATA TTT TAC TTC AGC<3´ and OdoCo1 rev 5´>CTT AAA TCC ATT GCA CTT TTC>3´ was designed as an odonate specific CO1 marker. The resulting 600bp long fragment is not located within the traditional barcode region. Polymerase chain reactions (PCRs) were carried out regarding the protocol of Abraham *et al*. [28] for ND1 and were as follows for CO1: 1× amplification buffer (20 mM Tris–HCl, pH 8.4; 50 mM KCl; Invitrogen), 2.5 mM MgCl_2_, 0.05 mM dNTPs, 0.5 pmol/l each primer, and 0.03 U/l *Taq* DNA polymerase under a thermal regime with 3 min initial denaturation at 95°C, followed by 35 cycles of 95°C for 30 s, 50°C for 40 s, 72°C for 40 s, and 2 min extension at 72°C. Cycle sequencing was performed using BigDye^®^ Terminator Cycle Sequencing Kit (Applied Biosystems) following a modified protocol. The amplified PCR products were sequenced on an ABI PRISM^®^ 310 Genetic Analyzer (Applied Biosystems) following the manufacter’s protocol using Sephadex Gel Filtration for purification (GE Healthcare).

For data analyses, DNASP version 4.0 [29] was employed to estimate parameters of haplotype (*h*) and nucleotide diversity (p) between generations of *O*. *coerulescens* from the CdV for all years investigated. Sequence divergence within a population as well as between all generations were calculated in MEGA 6 [30] according to the Kimura2-parameter model as described in Kimura [31]. Genetic differentiation in terms of fixation indices (*F*_*st*_) [32] was computed using ARLEQUIN (version 3.5.1.2; [33]). To visualize population structure over time, a mutational network based on statistical parsimony was generated using TCS (version 1.21; [34]). Default settings with a connection limit of 95% sequence similarity were kept.

### Microsatellite analyses

Based on the results of the mitochondrial sequence analyses, populations from specific years between 1989 and 2011 (Table 1) were selected and analyzed by means of microsatellite genotyping of six microsatellite loci according to Hadrys *et al*. [20]. Automated genotyping were carried out using the 500 ROX^™^ Size Standard (Applied Biosystems, GeneScan^™^) on an ABI PRISM^®^ 310 Genetic Analyzer (Applied Biosystems). For the allele size determination the GeneScan^®^ Analysis Software (Applied Biosystems) was used.

Genetic diversity parameters of *O*. *coeruelscens* populations between the sampled years were assessed by comparing allele frequencies, expected heterozygosity (H_E_), observed heterozygosity (H_O_), and allelic richness (A) for each locus via the software FSTAT (version 2.9.3.2; [35]). Inbreeding coefficients (Wright´s *F*-statistics) were calculated with *F*_IS_, based on observed heterozygosities as well as *F*_ST_ values based on expected heterozygosities with the software ARLEQUIN (version 3.5.1.2; [33]). *F*_*ST*_ values between all population pairs were additionally assessed and p-values were calculated using 1000 permutations. Deviations from Hardy-Weinberg equilibrium (HWE), the probabilities for conformity with Hardy-Weinberg proportions and linkage disequilibrium were calculated using the Markov chain method implemented in GENEPOP (version 4.0.10, [36]). STRUCTURE (version 2.1; [37]) was used to conduct a Bayesian multi-locus clustering as implemented in the software, based on a model clustering method for unlinked markers to estimate population structure by genotype data. Prior population definitions were ignored and all individuals were placed into *K* populations (with *K* unknown), which were clustered by distinctive allele frequencies. In accordance with the number of sampled years, runs with *K* values from one to seven were repeated 20 times using the admixture model with correlated frequencies. Runs were performed with a burn-in period of 10^5^ steps followed by 10^5^ Markov chain Monte Carlo replicates. The online version of STRUCTURE HARVESTER [38] was applied to estimate the most significant *K* out of the STRUCTURE run result files.

## Results

### Sequence analyses

**Fig 1:**
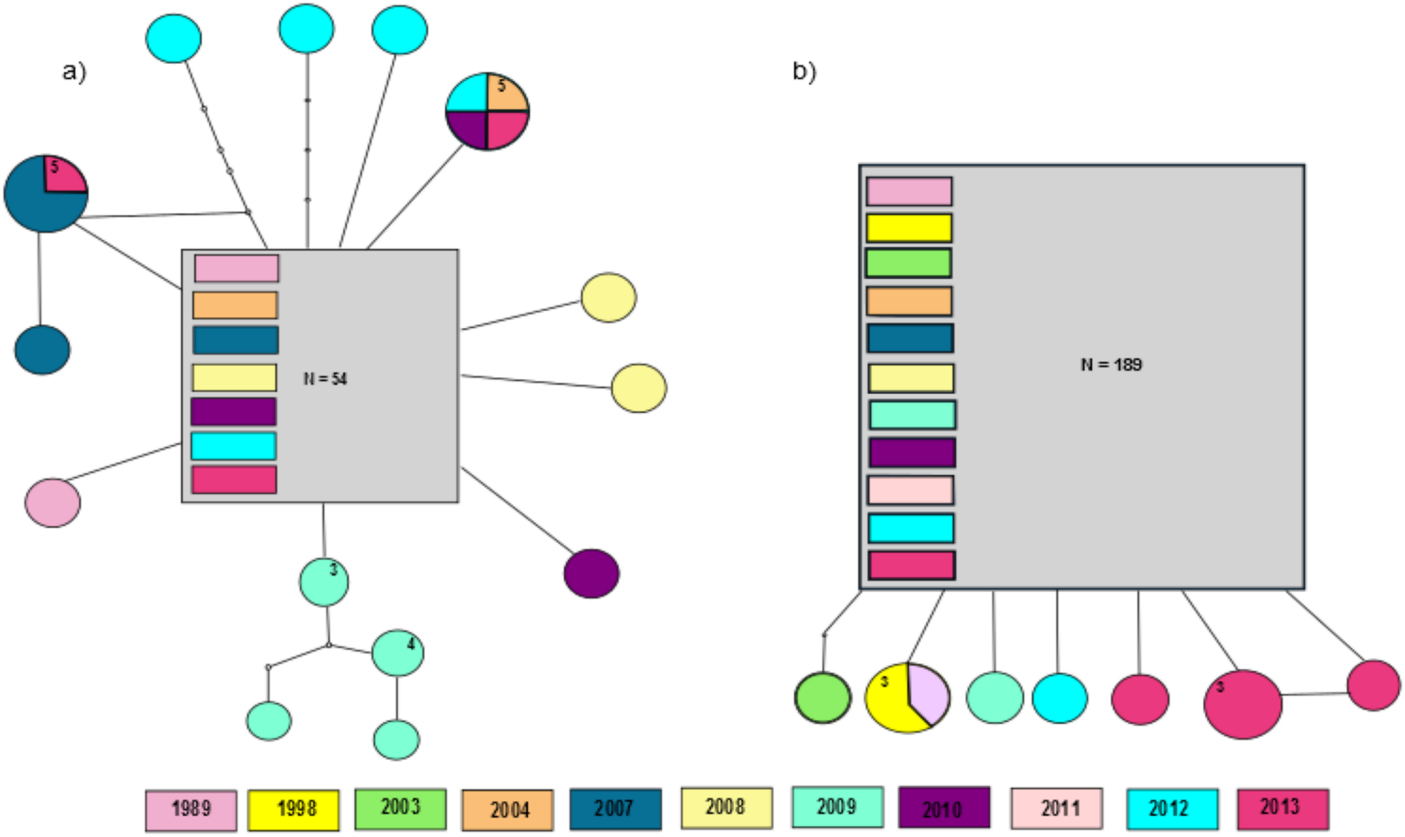
Mutational haplotype network for a) the CO1 and b) the ND1 gene fragment based on statistical parsimony. Shown are the genealogical relationships between the haplotypes of *O*. *coerulescens* in the years 1989 to 2013 at the Canal de Vergières. The considered ancestral haplotypes are depicted as rectangles, all other haplotypes as circles. Missing mutational steps connecting haplotypes are represented by small non-colored dots. Haplotypes connected by a single line differ in one mutational step. The size of the rectangle and circles correlates with haplotype frequency within each network. The different colors represent the different years, haplotype frequency is given in numbers if greater than one.

Sequencing of the ND1 gene fragment was successfully performed for 200 individuals. The resulting alignment was 509 bp long and contained seven variable nucleotide positions. For the CO1 fragment 38 variable sites were scored in a 601 bp long alignment including a subset of 84 individuals of *O*. *coerulescens* for the analyzed years. For ND1 eight haplotypes were detected in total (GenBank Accessions KY807703—KY807710), CO1 shows 15 haplotypes (GenBank Accessions KY807688—KY807702). The number of haplotypes per year varied between both markers. It ranges from one to four for ND1, with a maximum of three private haplotypes for the generation in the year 2013 (Fig 1). The ND1 haplotype network is dominated by one common haplotype, shown in 184 out of 200 individuals throughout all analyzed years (Fig 1).

The CO1 subset was envisaged to confirm the ND1 results, since former studies in odonates show mirrored results for both markers. However, in our data subset a significant higher genetic variation in terms of nucleotide substitutions was recorded regardless of the smaller sample size (see Table 2). Haplotype and nucleotide diversity were higher in general compared to the ND1 data. Most notably, all four CO1 haplotypes in 2009 occurred only in this year and no common haplotype was detected. Shared haplotypes were found between the years 2004, 2010, 2012 and 2013 and between the latter and 2007. Additionally, more “generation” specific private haplotypes were present for CO1 than for ND1 again regardless of the smaller sample size (see Fig 1).

**Table 2:** Genetic diversity parameters for the studied *O*. *coerulescens* populations over years. Sample size (*N*), number of haplotypes (H), haplotype diversity (*h*), nucleotide diversity in % (*π*) with standard deviation (SD) for both mitochondrial marker genes ND1 and CO1.

	ND1				CO1			
Population	*N*	H Total/private	*h*(±SD)	*π%*(±SD)	*N*	H Total/private	*h*(±SD)	*π*%(±SD)
**1989**	16	1/0	0.0	0.0	5	2/1	0.400 (±0.237)	0.067 (±0.039)
**1998**	24	1/0	0.0	0.0	--	--	--	--
**2004**	22	2/0	0.173 (±0.101)	0.034 (±0.020)	4	2/0	0.500 (±0.265)	0.083 (±0.044)
**2007**	12	1/0	0.0	0.0	5	3/1	0.700 (±0.218)	0.166 (±0.058)
**2008**	17	1/0	0.0	0.0	8	3/2	0.464 (±0.200)	0.083 (±0.039)
**2009**	17	2/1	0.118 (±0.010)	0.046 (±0.002)	10	4/4	0.778 (±0.091)	0.292 (±0.061)
**2010**	18	1/0	0.0	0.0	8	4/1	0.643 (±0.184)	0.125 (±0.044)
**2011**	21	2/0	0.095 (±0.071)	0.019 (±0.017)	--	--	--	--
**2012**	20	2/1	0.100 (±0.088)	0.020 (±0.017)	17	6/3	0.515 (±0.145)	0.624 (±0.355)
**2013**	18	4/3	0.471 (±0.130)	0.098 (±0.030)	15	2/0	0.343 (±0.128)	0.057 (±0.021)

### Microsatellite analyses

In total, 122 alleles were scored for the analyzed populations between 1989 and 2011 and the number of alleles per locus ranged from two to 17. Allelic diversity parameters are presented in Table 3. Allelic diversity (A) ranged from 5.5 to 9.7, averaged for the six analyzed loci. The highest value was found for the year 1998 (9.7) with a dramatic decline to 5.5 in the sampling period in 2007. Allelic richness (A_RC_), corrected for the smallest sample size, showed less variation ranging from 5.19 to 5.42 per population and locus for all studied years. The number of alleles scored within populations ranged from 33 in 2007 to 58 in 1998 with at least one private allele found in each year. The highest number of private alleles was scored in the year 1998 with a total number of eight. Observed heterozygosities across all loci ranged from 0.541 in 1989 to 0.770 in 2011 (see Table 3) showing a rising trend.

**Table 3:** Nuclear microsatellite diversity for the seven studied populations at the CdV from 1989 to 2011.

Population	*N alleles* total/private	A_locus_	A_Rc_	H_O_	H_E_	*F*_*IS*_	gd
**1989**	50/1	8.333	5.314	0.541	0.604	0.132	0.598 ± 0.349
**1998**	58/8	9.667	5.388	0.608	0.661	0.163	0.624 ± 0.397
**2004**	54/7	9.000	5.271	0.561	0.629	0.112	0.688 ± 0.430
**2007**	33/3	5.500	5.201	0.639	0.642	0.007	0.611 ± 0.381
**2008**	39/1	6.500	5.421	0.714	0.659	-0.257	0.878 ± 0.580
**2010**	40/3	6.667	5.190	0.714	0.651	-0.182	0.457 ± 0.357
**2011**	40/4	6.667	5.317	0.770	0.674	-0.185	0.687 ± 0.435

Number of alleles (N), number of alleles per locus (A_locus_), allelic richness averaged and corrected for sample size (A_RC_), observed (H_O_) and expected (H_E_) heterozygosities, inbreeding coefficients (*F*_*IS*_), average gene diversity over loci with standard deviation (gd).

All populations have at least one locus with significant deviation from the Hardy-Weinberg equilibrium (HWE). Generations in the years 1989, 1998 and 2004 displayed three to six loci with highly significant deviance from the HWE indicating a heterozygosity deficit. Generations after 2004 experienced a (less) significant heterozygous excess. The average of the observed heterozygosity is lower than expected from 1989 to 2007 and from thereon higher than expected, reaching values up to 0.77 in 2011 (see Table 3).

Allele frequencies for each locus per year are visualized in the supplementary data. For almost all loci different patterns in allele composition before and after 2004 can be found. In detail, for locus H04 and J04 different alleles dominated in the years 1989, 1998 and 2004 compared to the period from 2007 onwards. Furthermore, the number of alleles decreased after 2004 and rare alleles were lost, as most notable for locus F04 (S1 Fig).

Both *F*_*IS*_ and *F*_*ST*_ values were calculated, whereas the latter were assessed pairwise between all generations and years (see Table 4). *F*_*ST*_ values show significantly lower genetic differentiation between the years 1989, 1998 and 2004 when compared to the following years. Between more recent years (2007 to 2011) very low F_*ST*_ values were scored, but negative *F*_*IS*_ values (Table 3), indicating that individuals of these years might be less closely related than expected under a model assuming random mating but the overall genetic differentiation is low. The lowest *F*_*IS*_ value can be found in the population of 2008 with -0.257. For the years 1989, 1998 and 2004 positive and higher values can be observed in contrast. This implies a possible higher degree of inbreeding within these years as in the following years. The highest value (0.163) is scored in the year 1998 (Table 3) before the dredging event.

**Table 4:** Pairwise F_ST_ values of the *O*. *coerulescens* populations based on nuclear microsatellite analysis of six loci for the studied years at the *CdV*. Significant values (p < 0.05) are marked with asterisks. Lines indicate the split before and after the drought years.

	**1989**	**1998**	**2004**	**2007**	**2008**	**2010**	**2011**
**1989**	0						
**1998**	0.011*	0					
**2004**	0.012*	0.006	0				
**2007**	0.254*	0.234*	0.251*	0			
**2008**	0.262*	0.234*	0.260*	0.005	0		
**2010**	0.270*	0.234*	0.266*	0.004	0.007	0	
**2011**	0.259*	0.234*	0.255*	0.008	0.007	0.020*	0

**Fig 2:**
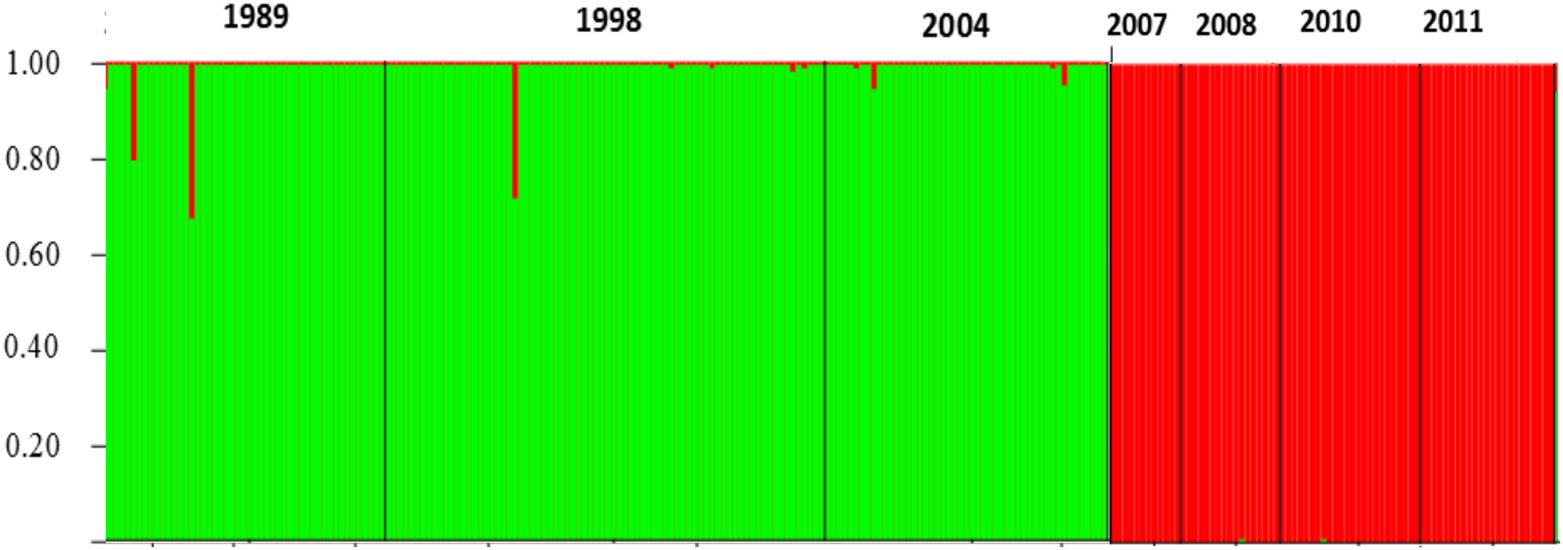
Bayesian analysis of the nuclear genetic structure of *O*. *coerulescens populations* at the CdV comparing seven years from 1989 to 2011 based on six microsatellite loci. Vertical lines represent a single individual and are partitioned into two colored segments indicating the estimated individual membership in each of the inferred cluster found by STRUCTURE.

To analyze the population structure via a multi-locus genotype data the model-based analysis was applied as implemented in STRUCTURE [37]. Microsatellite data of all individuals were clustered (*K* clusters); each cluster is characterized by a set of allele frequencies at each locus. Clusters were inferred without any predefined sets. Results revealed two main groups of individuals, containing populations from 1989, 1998 and 2004 in one group. Whereas populations from 2007 onwards were defined as the second genetic cluster (see Fig 2).

## Discussion

Genetic monitoring of species provides important and often novel information of their actual status. Long-term studies are able to detect population’s trends and may offer a background for management plans. Long-term projects were recently defined by a period of “at least 3 years” observation of the target species [39–42]. The obtained dataset of *O*. *coerulescens* at the Canal de Vergières covers a time frame of 24 years of monitoring. The application of a pair of population sensitive mtDNA markers and a panel of microsatellites revealed a significant cut and change in the population´s genetic make-up during the survey which was unexpectedly not caused by a direct anthropogenic impact and the following significant population decline.

### Genetic diversity

The mitochondrial markers ND1 and CO1 were successfully used in several odonate population genetic studies [e.g. 6, 21]. In this study they revealed an unexpected low genetic diversity for one of the largest known *O*. *coerulescens* populations in France, with only eight haplotypes for ND1 and 15 for CO1 in total (*N* = 200/84 individuals, respectively). The maximum number of haplotypes per generation was four (ND1) and six (CO1), respectively. Especially for ND1 higher variations are known for libellulid dragonflies demonstrated for example for *Trithemis arteriosa* [43] and *Crocothemis erythrea* [44] at comparable population sample sizes. Despite the smaller sample size of the CO1 dataset (used to proof the trend), a higher degree of variation can be recorded in terms of the number of (private) haplotypes as well as haplotype and nucleotide diversity. In other odonate studies CO1 mirrors ND1 data analyses [e.g. 6, 44]. In some years the two markers are not congruent, but the low genetic diversity is consistent between both datasets. The species´ environment might be a major driver of the genetic pattern since riverine odonates are highly restricted to their habitats and show less dispersal potential than other species adapted to a less stable habitat [45, 46]. As dispersal is one of the major drivers for genetic diversity [47] a higher diversity between and within populations can be assumed for good dispersers and generalists like *Trithemis* and *Crocothemis* (see above).

Despite this assumption of an *a priori* low(er) genetic variation for *O*. *coerulescens*, classic population genetic reasons for an induced loss of genetic diversity have to be taken into account. These are for instance a small population size, subsequent inbreeding, and genetic drift after a severe population decline [48–51]. They can be referred to anthropogenic influence to the natural habitat but also to biotic factors like extreme natural conditions.

### Population trends

For the years 1989 and 1998 adult densities were observed along the CdV in 20m long transects in July/ August three times a day between 11.00 a.m. to 16.00 p.m. Before dredging (1989), the average density was 28.5 individuals per transect whereas after this event only 7.6 individuals were found in 1998. The larval densities dropped from 35–40 individuals per square meter to 9–15 Ind/qm. All the very few other potential habitats in the surrounding (in 25km radius) had low population densities as well [52]. Despite the fact that population sizes were very high (except for 1998) overall variation and between the generations was low and the mitochondrial markers revealed no clear pattern of fluctuation in genetic metrics over the analyzed time period. Analyses of the microsatellite loci showed greater variation and a clear pattern that allows assumptions on population’s history, viability and trends.

Interestingly, the highest diversity regarding the number of alleles, private alleles and alleles per locus was scored in 1998, which was only two years after the dredging event, indicating that the dredging had not affected the population’s viability. This result is interestingly contradicting the population density data that indeed show a dramatic decline after the dredging. Both the larval and adult density dropped significantly. From 1989 to 1998 the population showed an increasing trend in terms of all analyzed genetic diversity parameters. After 1998 genetic diversity decreased, with a significant decline between 2004 and 2007. Individuals showed then the lowest allelic diversity (A_locus_ 5.5 from former 9.0 in 2004) and values remained low but stabilized in the following years up to 6.7 in 2011.

Observed heterozygosity increased over the whole time period, also from 1989 to 1998 regardless the demographic bottleneck in 1996. Between 2004 and 2007 though, the same genetic change could be detected. In sum, these three generations (1989, 1998 and 2004) displayed more loci with highly significant deviances from HWE as any other generations, indicating a heterozygosity deficit. Whereas generations after 2004 revealed less significant values and assuming a heterozygote excess, which is the typical sign of the genetic bottleneck [53, 54] we expected to see due to the dredging in 1996.

The split into two clusters based on microsatellite analyses before and after 2004, as well as strong genetic differentiation found between these generations by *F*_*ST*_ values supports the overall trend of a change in genetic diversity, possibly caused by a severe population size decline between the years 2004 and 2007 which could not be recovered from the surrounding satellite populations.

During this time period there was no anthropogenic impact on the canal, e.g. dredging. Instead a severe drought in 2003 was observed, followed by extreme hot and dry summers during 2004 to 2007. Although the CdV did not dry out, the water level was much lower than usual with no flow velocity (personal Information: Axel Wolff, Conservateur de la RNN des Coussouls de Crau). As the larvae of *O*. *coerulescens* are particularly dependent on specific water chemistry patterns, temperature, oxygen amount, sediment quality and flow velocity [55] the unusual high temperatures in these years with subsequent impact also on water chemistry associated factors may have influenced the survival of the larval population and consequently could have led to the observed decline. In contrast, for the year 2006 very low temperatures in the winter month and spring were recorded. Minimal average temperatures remained below freezing even until April in this year (personal Information: Axel Wolff, Conservateur de la RNN des Coussouls de Crau). Low temperatures in general do not affect most species’ larval development as they contain polyhydroxyethanols preventing them from freezing [55], but they have an impact on their life-cycle and emergence time during spring [56].

### Implementations for conservation

A combination of both, extreme and unlikely weather conditions is most likely the cause for the population decline between 2004 and 2007. In contrast, the clearing of the canal did not lead to a comparable dramatic effect. We assume that this local event could be compensated by (i) the surviving individuals and (ii) migration of closely related *O*. *coerulescens* individuals from surrounding ditches (compare Fig 2). This explains best the increase in allelic richness and heterozygosities from 1989 to 1998 after a demographic bottleneck. Adult densities in contrast decreased after the dredging, consequently a higher amount of inbreeding is expected (see *F*_*IS*_ values in Table 3). In the Crau we find a network of canals, from which the CdV with its largest population is most likely displaying the source-habitat under a metapopulation model. Indeed, by capture-mark-recapture analyses we could confirm that *O*. *coerulescens* individuals admix between the CdV and the closest other channel (Fosse de Poulanger, FdP) located in the Crau. Consequently, after a drastic local population depletion due to the dredging a recolonization by sink-individuals would not only slowly stabilize the population size, but also cause the unexpected gain of genetic diversity we observed between 1989 and 1998.

A change in climatic parameters affects the ecosystem on a larger scale. Extreme weather conditions like the recorded drought periods influence all freshwater habitats in the Crau. Hence, local population declines or even extinctions cannot be compensated out of a genetic metapopulation because all habitats of *O*. *coerulescens* in the area are affected. The change in the genetic make-up of the CdV population we detected after 2004 can only be explained by genetic drift due to changing ecological conditions and immigration of less related individuals to the former population. Consequently, we observed an increase in heterozygosity and *F*_*IS*_ values.

Small freshwater ecosystems in general and riverine ecosystems in special are increasingly prone to environmental change, especially in arid as well as developed landscapes [8]. The artificial channels in the Crau cannot recruit groundwater in consequence of the special soil conditions. But here lies a chance for conservation management in this sensitive ecosystem, as the water levels in artificial irrigation ditches can be managed. Consequently, a managed water supply in drought periods as conservational effort in order to protect the unique odonate fauna (and other aquatic insects) in this sensitive ecosystem would be highly desirable. However, for natural small creeks this opportunity does not exists. We assume that our findings translate to other sensitive, riverine dragonfly species, as well as riverine aquatic insects in the Mediterranean area.

In sum, only the long-term genetic monitoring data could reveal that the population of *O*. *coerulescens* at the CdV is rather affected by natural factors, like extreme weather conditions than by direct anthropogenic impacts. The population decline between 2004 and 2007 was not noticed by conventional surveys on this species abundance. The low genetic diversity despite a large population size and subsequent decrease of the ability to deal with environmental changes cannot be determined by traditional monitoring efforts. This stresses the value of long-term data sets on a regional scale for population genetics and conservation biology in order to understand the causal mechanisms on small scales behind global changes.

## Conclusion

Genetic analyses of data collected over a period of more than 20 years to the *Orthetrum coerulescens* at the CdV in Southern France revealed an overall low genetic diversity and a population decline between the years 2004 and 2007. Afterwards the population shows a stabilizing trend but is not reaching the amount of genetic variation from 1989. Surprisingly, the decline cannot be referred to the clearing of the canal in 1996, an anthropogenic event assumed to have great negative impact to the larval population of the target species. Instead, it is most likely that the populations’ decay was due to extreme climate conditions in 2004 and the following two years. Based on this local monitoring scheme we now can conclude that for the viability of this highly habitat specific insect species it might be important to study the effects of climate change to its riverine ecosystems on a long term basis and in a global setting.

## Supporting information

S1 Fig Relative allele frequencies per year investigated based on nuclear microsatellite analysis for the six chosen loci for the *O*. *coerulescens* populations at th*e Canal de Vergières*. Different alleles are represented by different colours.
(TIF)

S2 Fig Relative allele frequencies per year investigated based on nuclear microsatellite analysis for the six chosen loci for the *O*. *coerulescens* populations at th*e Canal de Vergières*. Different alleles are represented by different colours.
(TIF)
