# Supplementary figures and images for: Long-term genetic monitoring of a riverine dragonfly, Orthetrum coerulescens (Odonata: Libellulidae]: Direct anthropogenic impact versus climate change effects
Source: PLoS One. 2017 May 26;12(5):e0178014. doi: 10.1371/journal.pone.0178014 (PMC5446129; doi:10.1371/journal.pone.0178014)

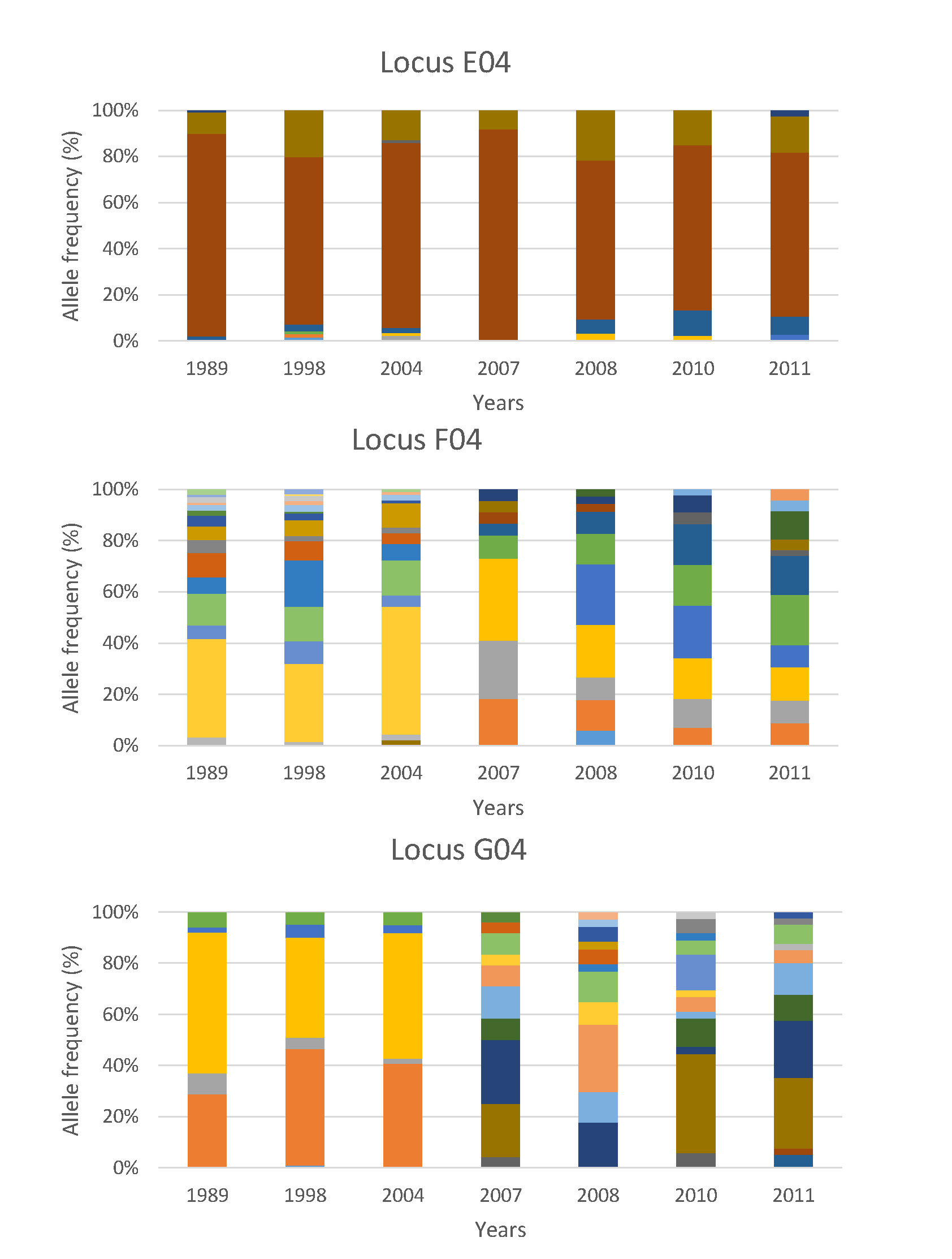

Supplement: S1 Fig — Different alleles are represented by different colours. (TIF) [file pone.0178014.s001.tif]

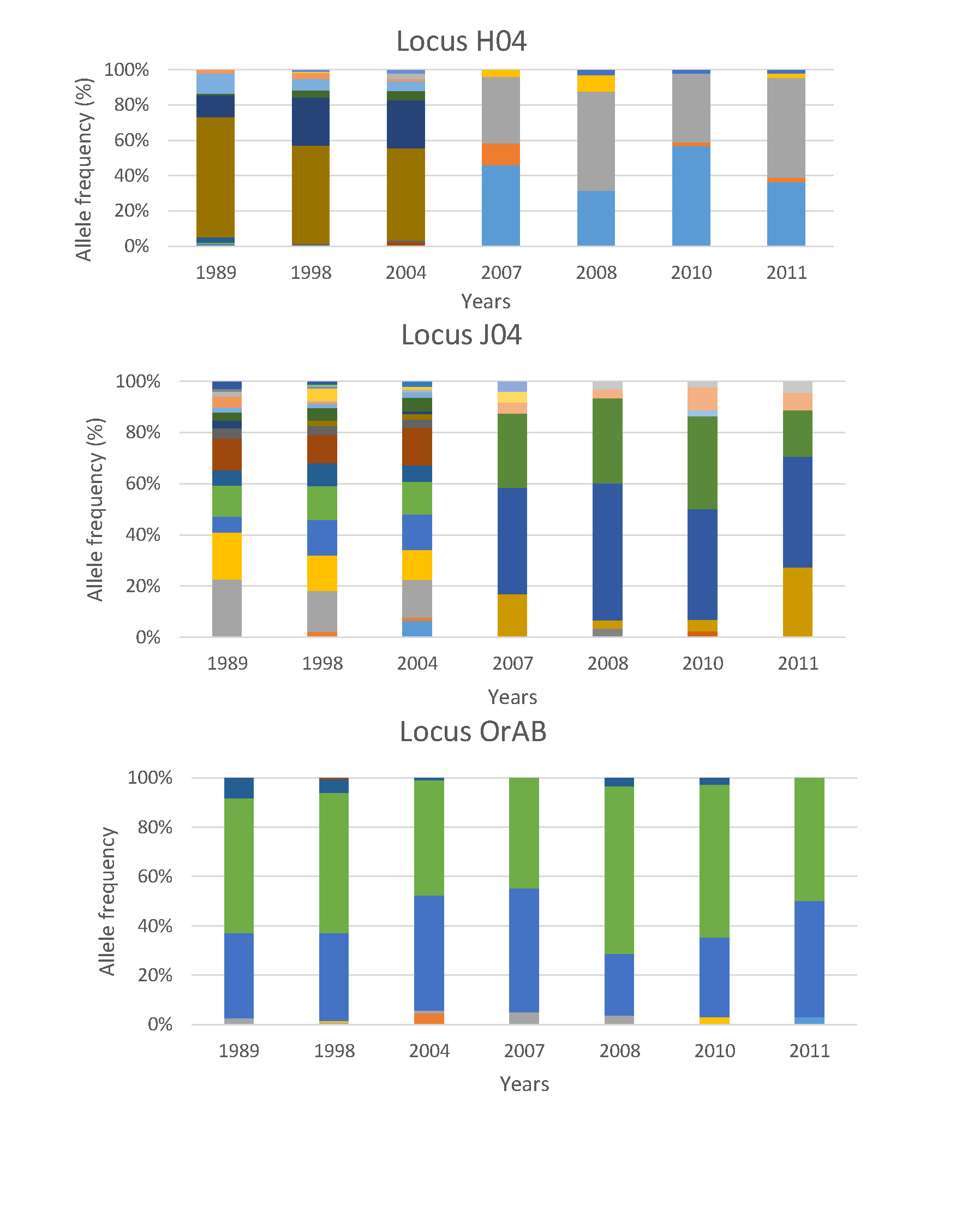

Supplement: S2 Fig — Different alleles are represented by different colours. (TIF) [file pone.0178014.s002.tif]
